# Supplementary material for: Evidence for Eocene aridification of the Atacama Desert’s hyperarid core
Source: Nat Commun. 2026 May 20;17:4520. doi: 10.1038/s41467-026-73422-4 (PMC13190850; doi:10.1038/s41467-026-73422-4)
Supplement: Supplementary file 1 — Supplementary Information [file 41467_2026_73422_MOESM1_ESM.pdf]

Supplementary Information 1

Evidence for Eocene aridification of the Atacama Desert's hyperarid core

Benedikt Ritter-Prinz [1*], Steven A. Binnie [1], Finlay M. Stuart [2], Derek Fabel [2], Richard Albert [3], Volker Wennrich [1], Tibor J. Dunai [1]

1 Institute of Geology & Mineralogy, University of Cologne, Germany

2 Isotope Geosciences Unit, Scottish Universities Environmental Research Centre, East Kilbride, UK

3 Frankfurt Isotope and Element Research Center (FIERCE), Goethe-Universität Frankfurt, Frankfurt, Germany

** Corresponding author: benedikt.ritter@uni-koeln.de*

Content of Supplementary Information:

1. Regional Setting
2. Sampling Site
3. Uplift history of the Coastal Cordillera

Supporting Datafiles

Supplementary_Datafile_1_TCN Data

Including all relevant terrestrial cosmogenic nuclide data.

Supplementary_Datafile_2_Tephra_Data

Including all relevant analytical data for dating of tephra deposits.

1. Regional Setting

The study area is located in the northern Atacama Desert within the Coastal Cordillera (Fig.1). The Coastal Cordillera consists of an eroded Jurassic to Early Cretaceous magmatic arc that is formed by extensive volcanic sequences and several plutons [1,2]. It reaches elevations between 900-1600m, bounded by a steep coastal cliff towards the Pacific Ocean in the west and the Central Depression in the east. As a separate morphotectonic unit, the Cordillera has been uplifting independently from the adjacent Central Depression since the Oligocene/Miocene [3,4]. As a consequence, since the Oligocene/Miocene the Coastal Cordillera has acted as a barrier for sediments originating from the Andes that became therefore deposited in the adjacent Central Depression [5]. The large-scale morphostructural units in the study area were formed during Late Oligocene–Early Miocene times when the coastal regions of northernmost Chile and southernmost

Peru were near sea level [6,7]. Thick sediment strata of the Azapa Formation can be linked to the synchronous deposits of the Lower Moquegue Formation, the lower part of the Altos de Pica Formation, the Tambores Formation; and the Calama Formation [8,9, and references therein]. These deposits are mainly composed of erosion products of the uplifting Andes to the east [10] and were predominantly deposited within the Central Depression, where they partially infilled basins and drainages towards the Pacific Ocean [6,11]. Sedimentation ceased around the Oligocene/Miocene boundary [7,11-13]. Differential uplift of the Coastal Cordillera subsequent the deposition resulted in the isolation of the Coastal Cordillera sedimentary systems.

The *minimum age* of the sediment sequence and surface of sampling site CH04/5 is constrained by the age of a dated ignimbrite layer at the top of a corresponding sediment sequence 8 km to the west, on the coastal cliff [12]. We additionally sampled the ignimbrite layer directly underlying the sampled sediment surface (sink). The age of the dated and published ignimbrite by Mortimer, et al. [12] is 21.8±0.3 Ma (K-Ar on biotite, 90.3% radiogenic ^{40}Ar [12], recalculated after [14] and [15]), indicating that this phase of sedimentation ended in the study area at the same time as the regional equivalents of the Moquegua and Azapa formations [7,12,13]. The sediment sequence dated by Mortimer, et al. [12] has been downfaulted along a NS-trending normal fault [11,12,16] and is now ~ 450 m lower than the investigated surface. Presumably, the same ignimbrite lies beneath the sampled sediment surface, outcropping on the southern rim of the Quebrada de Tiliviche. Maximum depositional ages based on the sampled ignimbrite layer directly underlying the sampled sediment surface (sink) is 25.19 ± 0.71 Ma (n = 3, TIL22-02) and 19.54 ± 0.92 Ma (n = 4, TIL22-03), further details are outlined below.

The thick sediments sourced from the east are intercalated with sabkha deposits, creating a karst landscape at the CH04-5 and PI18-01A sampling site. These are situated in a series of steep-walled salt karst depressions, mostly ~2 m deep. The salt karst is formed in old evaporites originally deposited in a saline mudflat environment near to or at sea-level [11]. The sampled sediment surface is now dissected by the incision of the Quebrada de Jazpampa and is bounded to the north by the deeply incised Quebrada de Tiliviche. The incision of the Quebradas occurred prior to 3.5 Ma and but after 6.4 Ma [17], initiated by overflowing of an endorheic lake formed at a topographic low of the Central Depression to the east and blocked by the higher elevated Coastal Cordillera [17]. The Quebrada de Jazpampa may be much younger, sourced from a temporary lake caused by the activity on the Pisagua fault (^{21}Ne 120 ka [11]).

2. Sampling Sites

<u>Samples from depositional surface at Pampa de Jaz (PI-03, PI-06, PI-07, CH04-5, PI18-001A):</u>

Sample and site descriptions for PI-03, PI-06 and PI-07 are documented in Dunai, et al. [11].

CH04-5: This site is located on the old sediment surface deposited around the Miocene/Oligocene boundary [11]. Samples were collected within an area of about 100 m^2 upstream of 19º33'50.373"S 70º7'3.871"W; elevation 933±1 m. The sampling area is located just upstream of the knickpoint where the $CaSO_4$ crust-covered valley floor gives way to the salt-karst depressions that dominate the valley floor further downstream. The fan deposits of the sub-catchments to the west of the site CH05/4 did not contain any vein quartz. Vein quartz similar to that sampled was found in sub-catchments located at least 4 km away to the south and south-east. The veneer of younger sediments on the sediment surface is very thin, with rarely more than one clast per m^2. A friable $CaSO_4$ crust covers the surface; with younger sedimentary clasts on top of the $CaSO_4$ crust. The quartz clasts are the residues of sheet flow deposits, with fine material subsequently removed by deflation and poly-mineral rock fragments destroyed by salt weathering. Salt weathering is promoted by the intense coastal fog (camanchaca) typical of the area [18,19]. Rare rock clasts are highly weathered and friable. Single-crystal vein-quartz clasts, such as those collected for this study, in contrast, are resistant to salt weathering. They are also the hardest material at the surface and therefore resist wind-erosion; the clasts collected show no significant signs of ventifaction. Following the 'born at the surface' model of Wells, et al. [20], stones of a desert pavement remain at the surface on an accretionary mantle of soil-modified dust and are successively lifted from the bedrock/original sediment surface. This process has been confirmed to be operational in the hyper-arid soils of the Atacama [21] and enables the continuous exposure of clasts at the surface of the alluvial fans. Any modification of the soil surface by deflation or gentle fluvial erosion would keep clasts on the surface [22]. In areas at ~1100 m elevation, 5 km to the north-east of site CH04/05, which are rarely affected by coastal fog, the surface is covered by a thick layer of flour-like $CaSO_4$ dust (several tens of cm), and devoid of rock clasts.

PI18-001A: This sampling site is located up to ~450 m further downstream of sampling site CH04-5 (19°33'36.73"S, 70° 7'6.29"W, 930 m a.s.l.) within the salt-karst area. Samples were collected from flat surfaces at the bottom of a karst depression (like Fig. S1E).

Coastal Cordillera Catchment PI06-1,2,4, PI17-04 :

PI06/1: Quartz clasts, associated with clasts of iron oxides and intermediate volcanics, on a friable $CaSO_4$ crust, approximately one quartz clast per m^2. Slope angle ~2º. 19º36'26.5"S 70º6'11.8"W; 1170 m a.s.l.

PI06/2: Quartz clasts, associated with clasts of iron oxides and boulders of intermediate volcanic rocks. Near-horizontal head of catchment (slope < 0.5º), approximately 10 to 20 quartz clast per m^2. 19º36'27.8"S 70º6'14.5"W; 1167 m a.s.l.

PI06/4: Local concentration of quartz clast on the flat top of a low hill. The hill has a continuous $CaSO_4$ crust cover like sites PI06/1&2. 19º36'27.5"S 70º6'18.7"W; 1180 m a.s.l.

Coastal Cordillera Catchment (PI17-004):

PI17-004: This sampling site is located up to 9 km further south of the studied catchment, within the same topographic high (19°40'32.24"S, 70° 4'0.11"W, 1195 m a.s.l.). We chose this sampling site because it is almost flat and represents the highest topographic point in the area. Clasts sampled from this surface can be considered as source rather than depositional environments and therefore may contain the highest concentrations of cosmogenic nuclides. The surface is covered by thick $CaSO_4$-rich soil. Quartz abundance is very low with only 1 clast per 4 m^2.

Tephra samples (TIL22-02, -03):

We sampled a thick outcropping tephra deposit on the rim of the Tiliviche Canyon. Two samples were taken from the lower and upper parts of this tephra and prepared for zircon U/Pb dating.

TIL22-02: S19°32’05.99’’ 869m

TIL22-03: S19°32’05.99’’ 877m

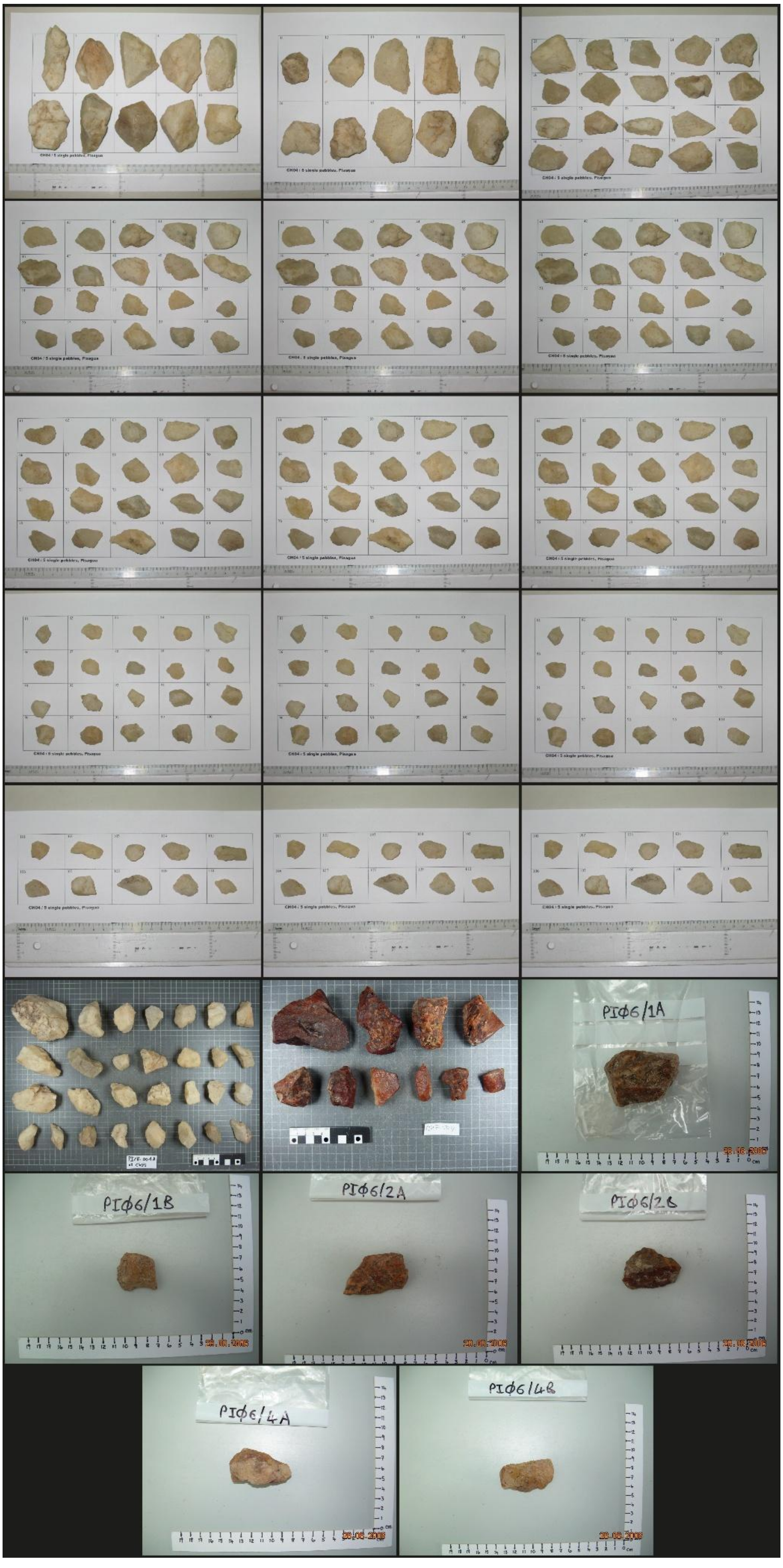

Supplementary Information Fig. S1: Sample Clast Image Compilation.

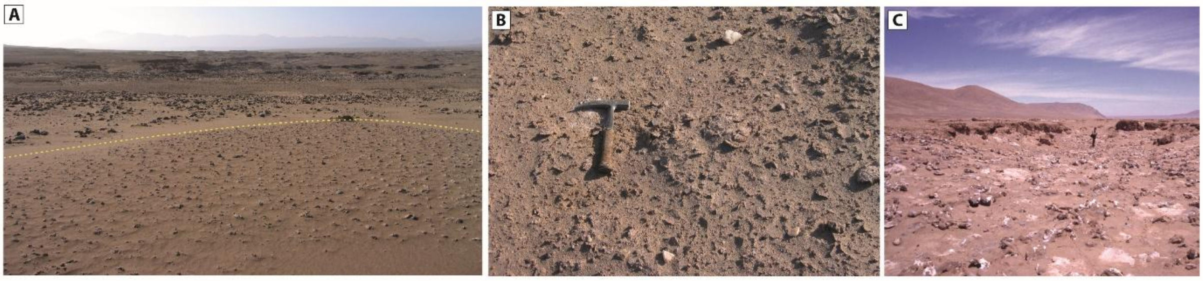

Supplementary Information Fig. S2: Jazpampa Surface Images: (A) View towards North from site CH04/5, samples were collected from the area in the foreground, to the south of the yellow stippled line. In the background, the salt-karst area is visible (C). (B) Detail of the surface at site CH04/5, showing the friable $CaSO_4$ crust and two quartz clasts sampled. The friable nature of the $CaSO_4$ crust is related to the frequent wetting by intense coastal fog. (C) Salt-karst pit at Site B [11].

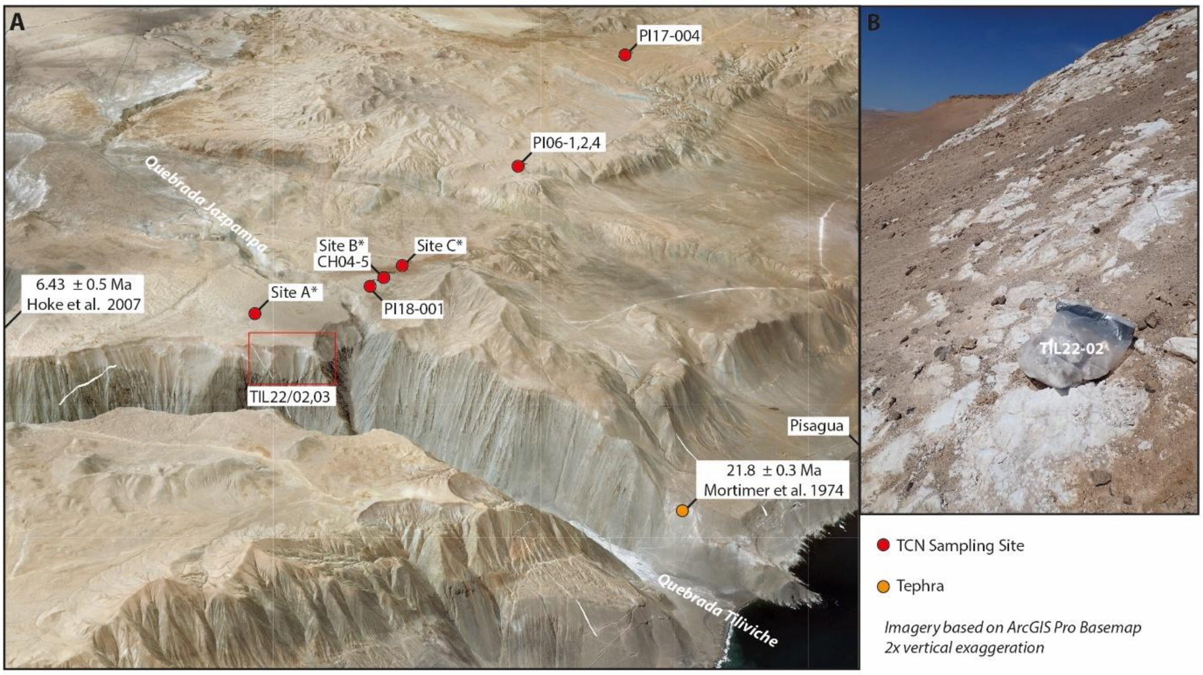

Supplementary Information Fig. S3 Tephra Location: (A) 3D overview map of the study area, created using ArcPro – WorldElevation3D/Terrain3D data, indicating all TCN sampling sites, published tephra ages and the sampled tephra site TIL22-02,03 marked with a red rectangle. Image is based on ArcGIS Pro Basemap with 2x vertical exaggeration. (B) Field image of TIL22-02.

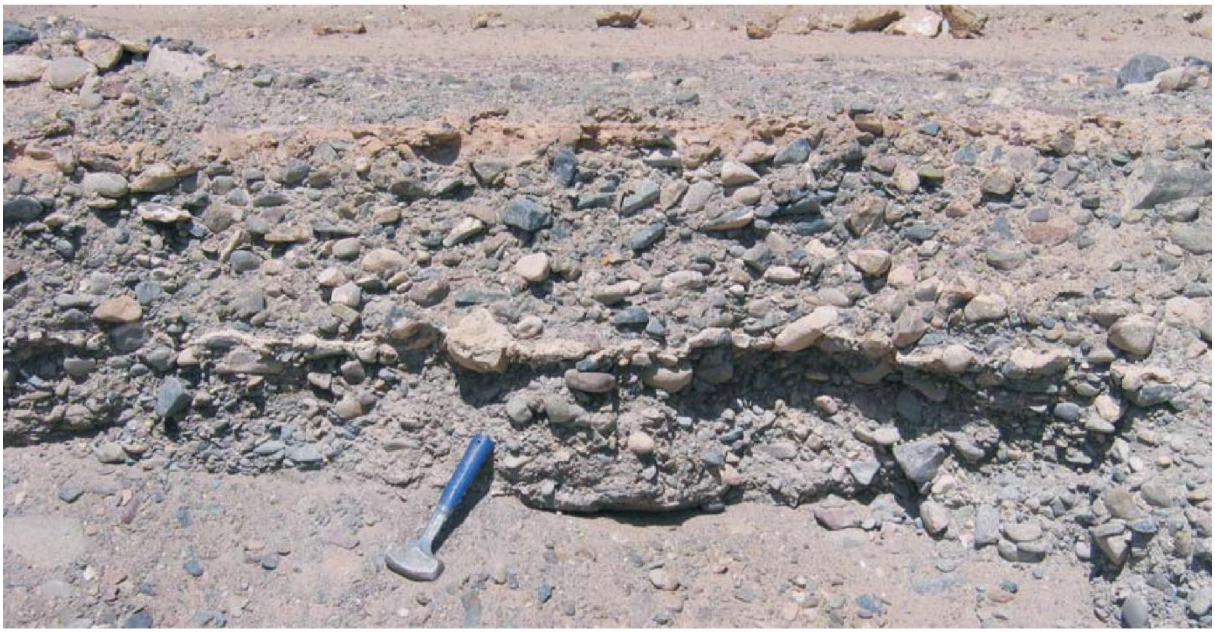

Supplementary Information Fig. S4: Sampling Site D from [11] - Outcropping Azapa Formation rounded gravels.

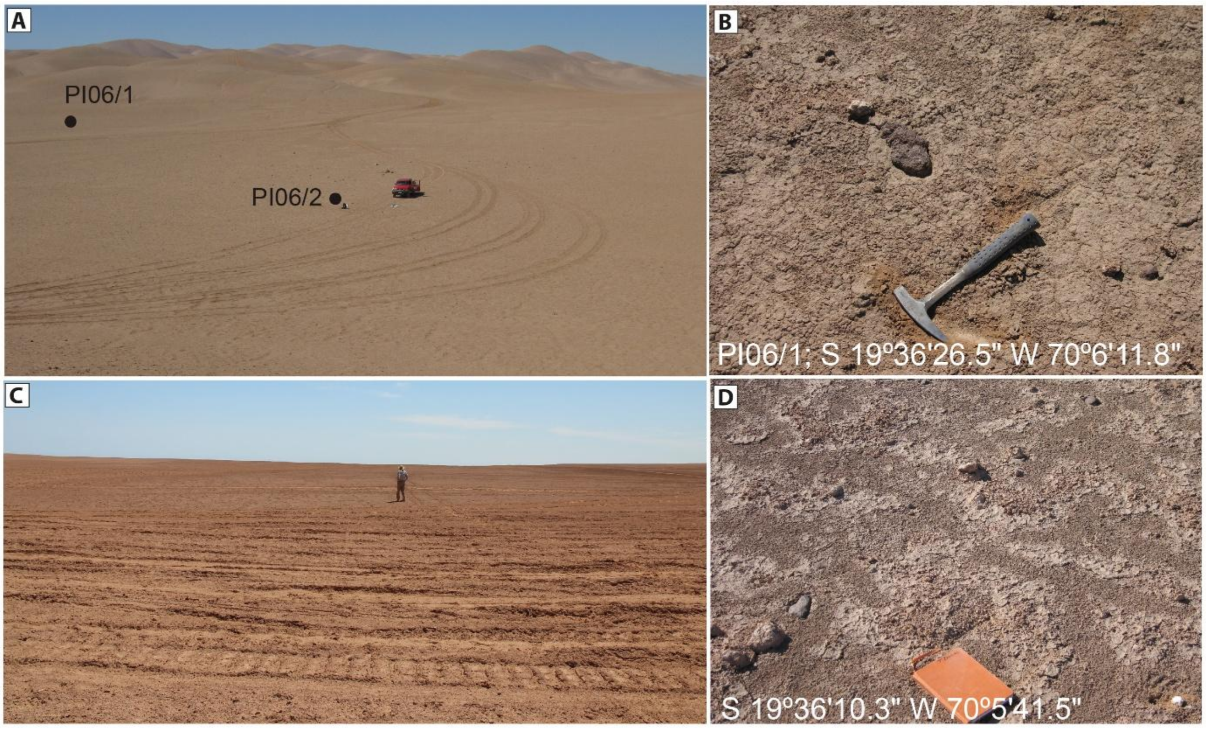

Supplementary Information Fig. S5: PI06- P17/4 Surfaces Images: (A) Overview of sampling sites PI06/1 and PI06/2. Picture taken from near site PI06/4 looking east. Site PI06/4 is located on the flat top of a gentle hill, similar to the one near PI06/1. The slope angle at site PI06/1 is ~2º, and <0.5º at site PI06/2. (B) Detail of sampling site PI06/1, showing clasts on a friable gypsum crust. The $CaSO_4$ crust is subtly patterned; the likely cause is swelling and shrinking of the crust in the near diurnal cycle of coastal fog wetting and subsequent desiccation. (C) Overview of sampling site PI17-04 looking to the west. (D) Detail of a gypsum crust at the head of the catchment immediately to the east of the catchment where samples PI06/1 & 2 were collected. This crust shows evidence of swelling (clast to the left of field book, book is 15 cm wide). Soil activity associated with the swelling and shrinking of the gypsum crust keeps the rock clasts on the surface and prevents their gradual disappearance under the gypsum dust that is continuously accumulated by atmospheric deposition.

3. Additional Results

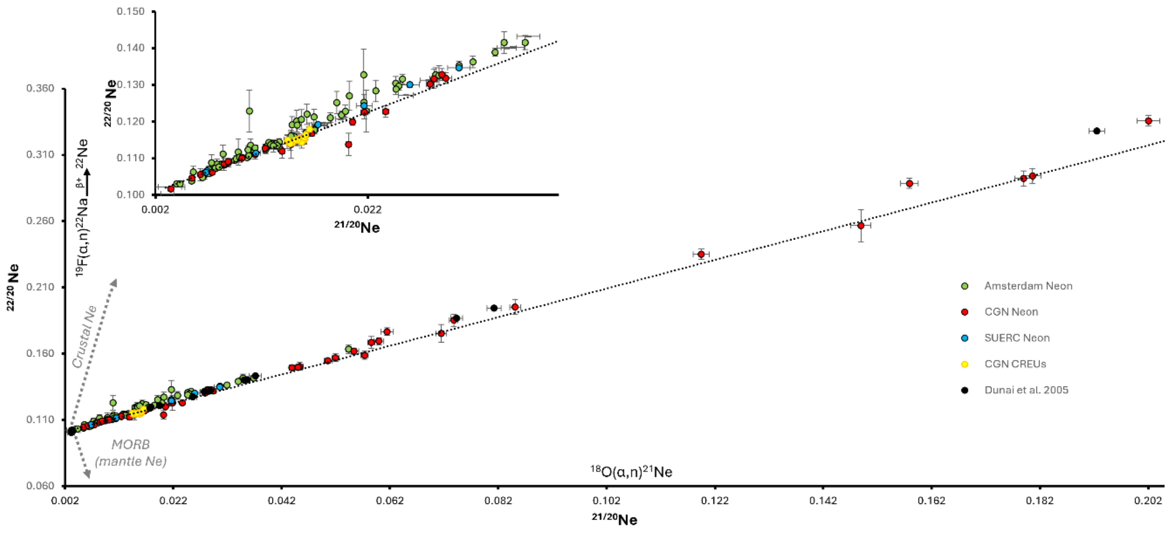

Supplementary Information Fig. S6: Neon Triple Isotope Plot: Neon Triple Isotope Plot ($^{22/20}Ne$ vs. $^{21/20}Ne$) for the samples given in Supplementary Datafile_1_TCN_Data. The dashed black line indicates the cosmogenic spallation line for neon after [23]. Trends of crustal and MORB Ne and reactions are taken from [24]. Samples measured at the VU Amsterdam are displayed in black, SUERC in green, Cologne in red and CREU-1[23] measured in Cologne in orange. Uncertainties are 1σ standard deviation. CREU-1, which has been analysed in an international laboratory comparison 60, consists of clasts taken from the study site described here (sample CH04/5, clasts 5, 6, 7, 8 and 13, see[23]); the non-atmospheric neon component in CREU-1 is essentially purely cosmogenic[23]. Nucleogenic neon, which could potentially mimic cosmogenic neon[25] can be excluded based on the low U and Th concentrations found in quartz from this location (<6 ppb; Dunai, et al. [11] supplementary data) and close to atmospheric isotopic ratios in neon released by crushing (Dunai, et al. [11] supplementary data).

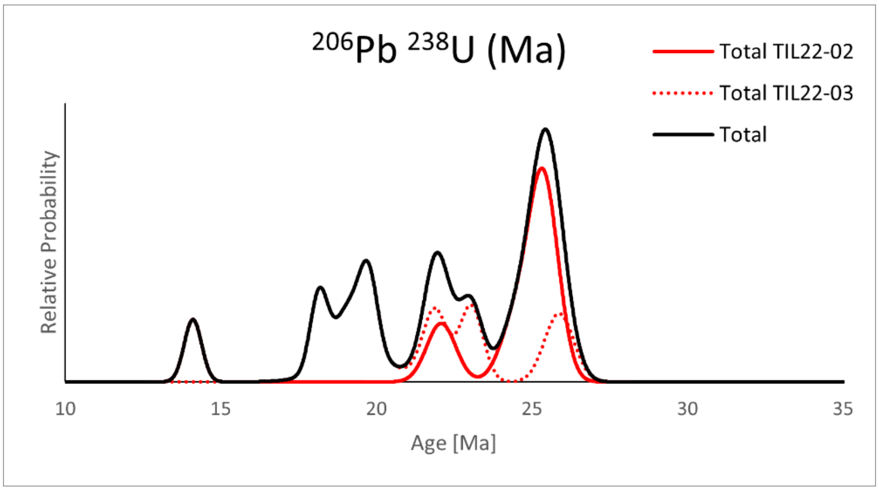

Supplementary Information Fig. S7: Tephra Age Compilation ^{206}Pb ^{238}U of sample TIL22-02 and TIL22-03. Data and uncertainties are reported in the Supplementary Datafile_2_Tephra_Data.

4. Uplift History of the Coastal Cordillera

The timing and rates of uplift in this area are poorly constrained [26]. With the evidence found at the sample site by Dunai, et al. [11], the assumption of an uplift rate of 40 m/Myr is reasonable.

Data on the paleo-topography of the Coastal Cordillera are scarce, as is direct evidence of uplift history and rates. In addition to the data and implications for the uplift history of the Coastal Cordillera in our study area presented in the regional setting section, there are other regional data that support the applied uplift-rate model and paleo topography. However, all these models and evidence are only approximate due to the lack of detailed temporally and spatially resolved uplift data. Neogene constraints of the uplift of the Coastal Cordillera are published, indicating recent and ongoing uplift, however, are largely limited to the Quaternary [27-30].

Tosdal, et al. [7] point out that the relief of the Coastal Cordillera in southernmost Peru was minimal prior to the Miocene, being close to sea level in the mid-Tertiary. Additionally, data from Noble, et al. [6] indicates that the 'coastal region was at a low elevation during latest Oligocene time" (Upper Moquegua Formation, time equivalent to Azapa Formation, see also Tosdal, et al. [7]). According to Armijo, et al. [31] and Hartley, et al. [3], the uplift and delineation of the Coastal Cordillera as a discrete morphotectonic unit has created accommodation space in the adjacent Central Depression since at least the Late Oligocene/Miocene time. For simplicity and due to the lack of sufficient detailed data on the uplift history, we used the Oligocene/Miocene boundary (23 Ma) for our uplift correction.

However, in order to test different rates of uplift and different topographies of the Coastal Cordillera, we performed a sensitivity test to capture the potential effect of these factors on our dataset. Assuming faster (60 m/Ma until ca. 15.66 Ma) or slower (20 m/Ma until ca. 46 Ma) uplift, exposure ages/durations can change the data (including uncertainties) by a maximum of 7.3% towards older ages or by a maximum of 15.4% towards younger ages. Data can be found in the Supplementary_Datafile_1_TCN_Data.

References

1 Riquelme, R., Martinod, J., Herail, G., Darrozes, J. & Charrier, R. A geomorphological approach to determining the Neogene to Recent tectonic deformation in the Coastal Cordillera of northern Chile (Atacama). *Tectonophysics* **361**, 255-275 (2003). https://doi.org/10.1016/s0040-1951(02)00649-2

2 Vásquez, P. & Sepúlveda, F. A. in *Serie Geología Básica 162-163* (Servicio Nacional de Geología y Minería, Santiago, 2013).

3 Hartley, A. J. *et al.* Development of a continental forearc: A Cenozoic example from the Central Andes, northern Chile. *Geology* **28**, 331-334 (2000).

4 Juez-Larre, J., Kukowski, N., Dunai, T. J., Hartley, A. J. & Andriessen, P. A. M. Thermal and exhumation history of the Coastal Cordillera arc of northern Chile revealed by thermochronological dating. *Tectonophysics* **495**, 48-66 (2010). https://doi.org/10.1016/j.tecto.2010.06.018

5 Mortimer, C. Drainage evolution in the Atacama desert of northernmost Chile. *Rev. Geol. Chile* **11**, 2-28 (1980).

6 Noble, D. C., Sebrier, M., Megard, F. & McKee, E. H. Demonstration of two pulses of Paleogene deformation in the Andes of Peru. *Earth Planet. Sci. Lett.* **73**, 345-349 (1985).
7 Tosdal, R. M., Clark, A. H. & Ferrar, E. Cenozoic polyphase landscape and tectonic evolution of the Cordillera Occidental, southernmost Peru. *Geol. Soc. Am. Bull.* **95**, 1318-1332 (1984).
8 Hartley, A. J. & Evenstar, L. Cenozoic stratigraphic development in the north Chilean forearc: Implications for basin development and uplift history of the Central Andean margin. *Tectonophysics* **495**, 67-77 (2010). https://doi.org/10.1016/j.tecto.2009.05.013
9 Evenstar, L. *et al.* Geomorphology on geologic timescales: Evolution of the late Cenozoic Pacific paleosurface in Northern Chile and Southern Peru. *Earth-Science Reviews* (2017).
10 Garcia, M. & Herail, G. Fault-related folding, drainage network evolution and valley incision during the Neogene in the Andean Precordillera of Northern Chile. *Geomorphology* **65**, 279-300 (2005). https://doi.org/10.1016/j.geomorph.2004.09.007
11 Dunai, T. J., Lopez, G. A. G. & Juez-Larre, J. Oligocene-Miocene age of aridity in the Atacama Desert revealed by exposure dating of erosion-sensitive landforms. *Geology* **33**, 321-324 (2005). https://doi.org/doi.org/10.1130/g21184.1
12 Mortimer, C., Ferrar, T. E. & Saric, N. K-Ar ages from Tertiary lavas of the northernmost Chilean Andes. *Geologische Rundschau* **63**, 484-490 (1974).
13 Wörner, G., Uhlig, D., Kohler, I. & Seyfried, H. Evolution of the West Andean Escarpment at 18ºS (N.Chile) during the last 25 Ma: uplift, erosion and collapse through time. *Tectonophysics* **345**, 183-198 (2002).
14 Böhlke, J. *et al.* Isotopic compositions of the elements, 2001. *Journal of Physical and Chemical Reference Data* **34**, 57-67 (2005).
15 Renne, P. R., Balco, G., Ludwig, K. R., Mundil, R. & Min, K. Response to the comment by WH Schwarz et al. on "Joint determination of 40K decay constants and 40Ar∗/40K for the Fish Canyon sanidine standard, and improved accuracy for 40Ar/39Ar geochronology" by PR Renne et al.(2010). *Geochimica et Cosmochimica Acta* **75**, 5097-5100 (2011).
16 Allmendinger, R. W., Gonzalez, G., Yu, J., Hoke, G. & Isacks, B. Trench-parallel shortening in the Northern Chilean Forearc: Tectonic and climatic implications. *Geological Society of America Bulletin* **117**, 89-104 (2005). https://doi.org/Doi 10.1130/B25505.1
17 Kirk-Lawlor, N., Jordan, T. L., Rech, J. A. & Lehman, S. B. Late Miocene to Early Pliocene paleohydrology and landscape evolution of Northern Chile, 19° to 20° S. *Palaegeography Palaeoclimatology Palaeoecology* **387**, 76-90 (2013).
18 Cáceres, L. *et al.* Relative humidity patterns and fog water precipitation in the Atacama Desert and biological implications. *Journal of Geophysical Research* **112** (2007). https://doi.org/10.1029/2006jg000344
19 Cereceda, P., Larrain, H., Osses, P., Farias, M. & Egana, I. The climate of the coast and fog zone in the Tarapaca Region, Atacama Desert, Chile. *Atmospheric Research* **87**, 301-311 (2008). https://doi.org/10.1016/j.atmosres.2007.11.011
20 Wells, S. G., McFadden, L. D., Poths, J. & Olinger, C. T. Cosmogenic ^{3}He surface exposure dating of stone pavements. *Geology* **23**, 613-616 (1995).
21 Wang, F. *et al.* Beryllium-10 concentrations in the hyper-arid soils in the Atacama Desert, Chile: Implications for arid soil formation rates and El Niño driven changes in Pliocene precipitation. *Geochimica et Cosmochimica Acta* **160**, 227-242 (2015). https://doi.org/http://dx.doi.org/10.1016/j.gca.2015.03.008
22 Ritter, B. *et al.* Neogene fluvial landscape evolution in the hyperarid core of the Atacama Desert. *Scientific Reports* **8**, 13952 (2018). https://doi.org/10.1038/s41598-018-32339-9
23 Vermeesch, P. *et al.* Interlaboratory comparison of cosmogenic Ne-21 in quartz. *Quaternary Geochronology* **26**, 20-28 (2015). https://doi.org/doi.org/10.1016/j.quageo.2012.11.009
24 Niedermann, S. Cosmic-ray-produced noble gases in terrestrial rocks: dating tools for surface processes. *Reviews in Mineralogy and Geochemistry* **47**, 731-784 (2002).
25 Hetzel, R. *et al.* ^{21}Ne versus ^{10}Be and ^{26}Al exposure ages of fluvial terraces: the influence of crustal Ne in quartz. *Earth Planet. Sci. Lett.* **201**, 575-591 (2002).

26 Evenstar, L., Mather, A. E. & Hartley, A. Using spatial patterns of fluvial incision to constrain continental-scale uplift in the Andes. *Global and Planetary Change* **186**, 103119 (2020).
27 Binnie, A. *et al.* Accelerated late quaternary uplift revealed by 10 Be exposure dating of marine terraces, Mejillones Peninsula, northern Chile. *Quaternary Geochronology* **36**, 12-27 (2016).
28 Victor, P., Sobiesiak, M., Glodny, J., Nielsen, S. N. & Oncken, O. Long-term persistence of subduction earthquake segment boundaries: Evidence from Mejillones Peninsula, northern Chile. *Journal of Geophysical Research-Solid Earth* **116**, B02402 02410.01029/02010jb007771 (2011). https://doi.org/B02402 10.1029/2010jb007771
29 Ortlieb, L. *et al.* Coastal deformation and sea-level changes in the northern Chile subduction area (23ºS) during the last 330 ky. *Quaternary Sci. Rev.* **15**, 819-831 (1996).
30 González-Alfaro, J. *et al.* Abrupt increase in the coastal uplift and earthquake rate since~ 40 ka at the northern Chile seismic gap in the Central Andes. *Earth and Planetary Science Letters* **502**, 32-45 (2018).
31 Armijo, R., Lacassin, R., Coudurier-Curveur, A. & Carrizo, D. Coupled tectonic evolution of Andean orogeny and global climate. *Earth-Science Reviews* **143**, 1-35 (2015).
