# Supplementary material for: Evidence for Eocene aridification of the Atacama Desert’s hyperarid core
Source: Nat Commun. 2026 May 20;17:4520. doi: 10.1038/s41467-026-73422-4 (PMC13190850; doi:10.1038/s41467-026-73422-4)
Supplement: Supplementary file 2 — Description of Additional Supplementary Files [file 41467_2026_73422_MOESM2_ESM.pdf]

Description of Additional Supplementary Files

File Name: Supplementary Data 1

Description: The supplementary data file compiles all cosmogenic nuclide data and data evaluation used in this study.

File Name: Supplementary Data 2

Description: The supplementary data file compiles all U-Pb LA-ICP-MS data of our tephra geochronology.
